# Supplementary material for: Relaxed Substrate Specificity in Qβ Replicase through Long-Term In Vitro Evolution
Source: Life (Basel). 2021 Dec 26;12(1):32. doi: 10.3390/life12010032 (PMC8778257; doi:10.3390/life12010032)
Supplement: Supplementary file 1 [file life-12-00032-s001.zip › life-1505940-supplementary.pdf]

Supplementary Materials

Relaxed substrate specificity in RNA replicase through long-term in vitro evolution

Kohtoh Yukawa[a], Ryo Mizuuchi[b], and Norikazu Ichihashi[a, c, d]

[a] Department of Life Science, Graduate School of Arts and Science, The University of Tokyo, 3-8-1 Komaba, Meguro-ku, Tokyo, 153-8902, Japan

[b] JST, PRESTO, Kawaguchi, Saitama 332-0012, Japan

[c] Komaba Institute for Science, The University of Tokyo, 3-8-1 Komaba, Meguro, Tokyo 153-8902, Japan

[d] Research Center for Complex Systems Biology, Universal Biology Institute, The University of Tokyo, 3-8-1 Komaba, Meguro, Tokyo 153-8902, Japan

Table S1. Nonsynonymous mutations fixed in the RNA polymerase encoded by each RNA.

Position	Original	RNA1	RNA2	RNA3
2	S	P	P	P
11	L	L	L	F
25	V	A	A	A
91	V	V	V	A
124	G	G	C	G
147	G	D	D	D
167	G	S	S	S
208	K	K	E	E
282	I	M	M	M
293	P	R	R	R
351	S	S	P	S
366	C	R	R	R
447	Y	Y	Y	H
448	L	L	R	L
459	Q	Q	R	Q
460	R	H	R	H
501	R	Q	Q	Q
535	S	F	F	F
549	N	D	D	D
556	S	P	P	P
600	P	S	S	S

Table S2 All mutations fixed in the RNAs

position	Original	RNA1	RNA2	RNA3	position	Original	RNA1	RNA2	RNA3
40	G	A	A	A	625	A	A	A	C
46	A	G	G	G	630	A	A	G	A
49	A	A	G	A	668	G	A	A	A
53	A	A	G	A	727	G	A	A	A
72	C	C	C	T	750	G	A	G	A
78	T	T	A	T	850	A	A	G	G
86	C	C	T	T	861	T	C	C	C
114	T	T	C	C	1074	A	G	G	G
116	A	A	G	A	1106	C	G	G	G
129	-	-	-	A	1279	T	T	C	T
141	G	G	G	A	1324	T	C	C	C
165	-	T	T	T	1530	A	T	T	T
166	-	C	C	C	1567	T	T	T	C
167	-	T	T	T	1571	T	T	G	T
168	-	A	A	A	1604	A	A	G	A
169	-	G	G	G	1607	G	A	G	A
170	-	A	A	A	1623	T	C	C	C
174	C	T	T	T	1730	G	A	A	A
193	A	G	G	G	1832	C	T	T	T
210	T	C	C	C	1836	G	T	T	T
225	A	A	G	A	1873	A	G	G	G
228	T	C	C	C	1894	T	C	C	C
232	T	C	C	C	1902	C	T	T	T
259	C	C	C	T	1920	T	C	C	C
302	T	C	C	C	1930~2019		-	-	-
500	T	T	T	C	2026	C	T	T	T
564	G	G	G	A	2040	A	G	G	G
598	G	G	T	G	2071	G	A	A	A

5-ethynyl-2'-deoxyuridine 5'-triphosphate
(EdUTP)

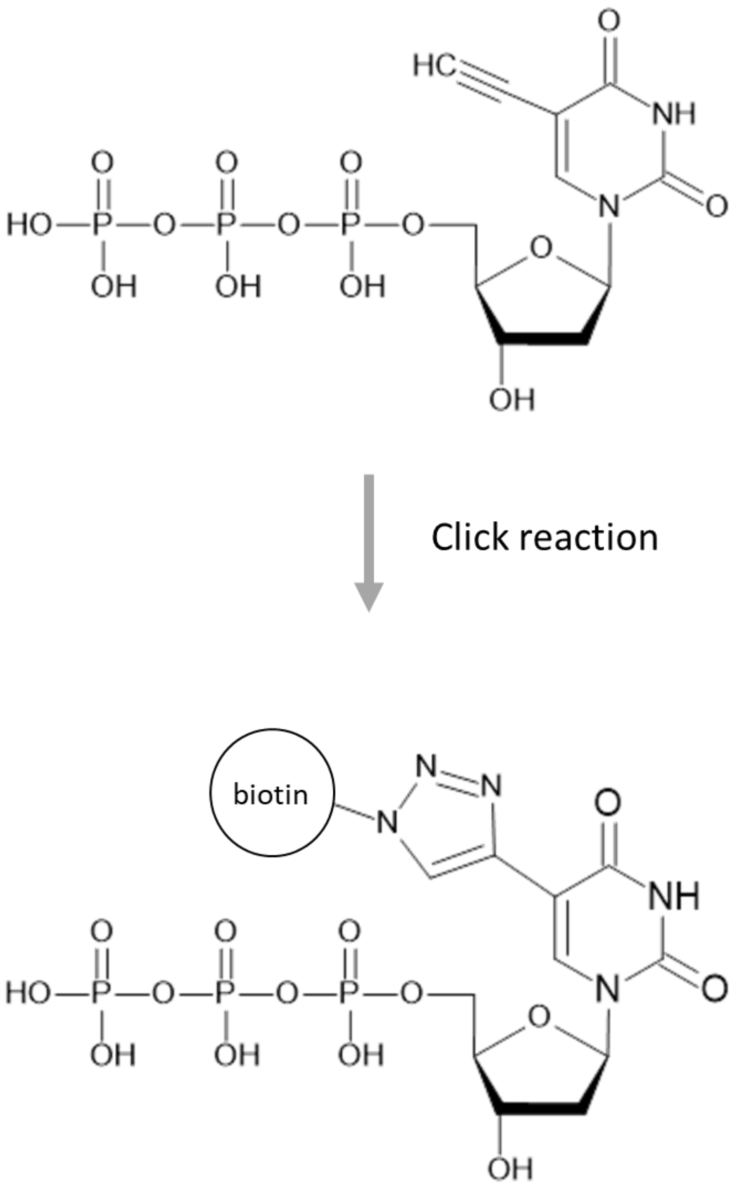

Figure S1. Chemical structure of EdUTP and the product of click reaction.

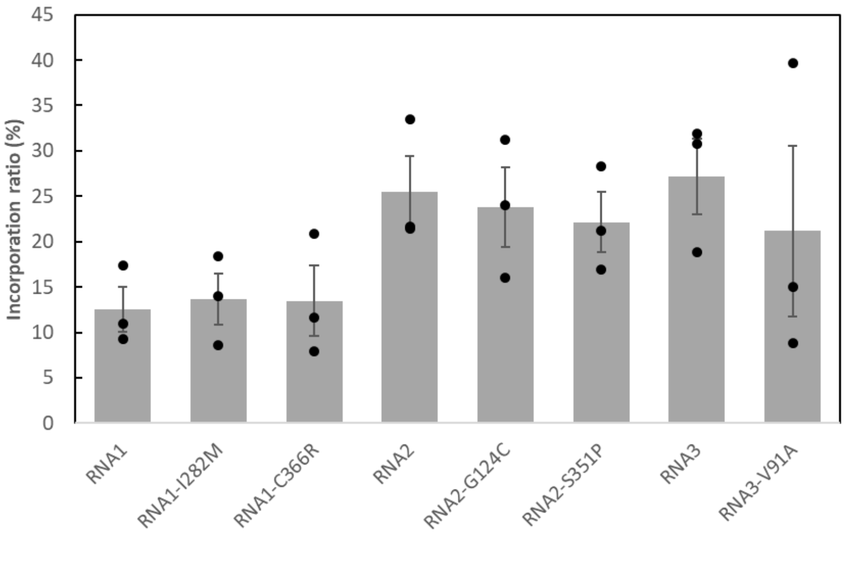

Figure S2. EUTP incorporation efficiency of the reverse mutants.

Among the mutations in each RNA, we created reverse mutants for those close to the NTP tunnel, and the EUTP incorporation ratio was measured in the same way as in Fig. 2. The results for RNA1–3 are shown for comparison. The names of the reverse mutants are shown as "name of the original RNA"-"name of the mutation that is reversed" (e.g., RNA1-I283M). Error bars represent standard error (N=3).
